# Jingui Shenqi Pills Regulate Bone-Fat Balance in Murine Ovariectomy-Induced Osteoporosis with Kidney Yang Deficiency

**DOI:** 10.1155/2020/1517596

**Published:** 2020-09-04

**Authors:** Qi Shang, Wenhua Zhao, Gengyang Shen, Xiang Yu, Zhida Zhang, Xuan Huang, Weicheng Qin, Guifeng Chen, Fuyong Yu, Kai Tang, Honglin Chen, Juanmin Li, De Liang, Jingjing Tang, Xiaobing Jiang, Hui Ren

**Affiliations:** ^1^Guangzhou University of Chinese Medicine, Guangzhou 510405, China; ^2^Lingnan Medical Research Center of Guangzhou University of Chinese Medicine, Guangzhou 510405, China; ^3^Department of Spinal Surgery, The First Affiliated Hospital of Guangzhou University of Chinese Medicine, Guangzhou 510405, China

## Abstract

Jingui Shenqi Pills (JGSQP) have been a staple of traditional Chinese medicine for thousands of years, used primarily as a treatment for kidney yang deficiency (KYD). *In vitro* analyses of JGSQP revealed strong induction of osteogenic differentiation and inhibition of adipogenic differentiation in bone-marrow-derived mesenchymal stem/stromal cells. However, the mechanisms by which JGSQP regulate the bone-fat balance in murine ovariectomy-induced osteoporosis with KYD have not been reported. *Materials and Methods*. Two-month-old female C57BL/6 mice were divided randomly into three groups: those receiving a sham operation (Sham); those undergoing bilateral ovariectomy and selection of KYD syndrome (Model); and those subjected to both bilateral ovariectomy and KYD syndrome selection for 8 weeks, followed by JGSQP treatment for 4 weeks (JGSQP). In the Sham and Model groups, mice were given the same dose of distilled water orally for 4 weeks. Animals from all three groups were euthanised at the 12th week. Vertebral microarchitecture and histomorphology were examined by micro-CT and H&E staining, respectively. In addition, we examined the mRNA expression of *Akt*, *Wnt10b*, *Osterix (Osx)*, *Fndc5*, *PPARγ*, and *Fabp4*, as well as the protein of AKT, phosphorylation-AKT (p-AKT), BMP2, COL1A1, and FNDC5. *Results*. JGSQP treatment improved bone microarchitecture and mitigated histomorphological damage relative to the Model group. The osteoblast number (Ob.N/BS) and area (Ob.S/BS) were increased, whereas adipocyte number (adipocyte/tissue area) and area (adipocyte area/tissue area) were decreased in the JGSQP group. JGSQP treatment reduced the mRNA expression of *Akt* and adipogenesis-related genes (*Fndc5*, *PPARγ*, and *Fabp4*) while promoting osteogenesis-related genes (*Wnt10b* and *Osx*) mRNA expression. Additionally, the expression of p-AKT, BMP2, and COL1A1 proteins was increased and FNDC5 protein expression was decreased after JGSQP treatment. *Conclusions*. JGSQP treatment reversed murine ovariectomy-induced osteoporosis with KYD by controlling bone-fat balance via AKT pathway.

## 1. Introduction

Postmenopausal osteoporosis (PMOP) is brought on by a dramatic drop in oestrogen among postmenopausal women, leading to decreases in bone mass and density and an increase in the risk of fragility fracture [1]. With the gradual ageing of the population throughout the world, the incidence of PMOP and associated fractures continues to increase annually, posing a serious threat to public health. Worldwide, 30–50% of postmenopausal women have osteoporosis [2], with significant differences based on ethnicity and nationality. White women aged >50 years were shown to have a 50% lifetime risk of fragility fracture [1], and ∼40% of postmenopausal women in Europe and the United States are diagnosed with osteoporosis [3]. Disability rates of up to 50% and a mortality rate of 20% have been reported in association with fragility fracture [4]. Medical expenses associated with fragility fractures in China are predicted to be as high as 163 billion yuan in 2050 [5]. Traditional Chinese medicine (TCM) has been shown to offer unique advantages in the treatment of PMOP, although the mechanisms underlying its therapeutic benefits remain poorly understood, which limits its clinical application.

TCM practitioners hold the view that kidney dominates bone, and kidney yang deficiency (KYD) is a key pattern underlying osteoporosis treatment based on the TCM practice of syndrome differentiation [6, 7]. Modern medical studies have shown that KYD is characterised by multiple disorders, including hypofunction of the pituitary–adrenal axis, decreases in antioxidation capacity, hypoimmunity, and age-related conditions [8, 9], all of which are related closely to osteoporosis [10–13]. Furthermore, analysis of serum taken from patients with PMOP revealed decreased osteogenic differentiation and mineralisation in a human osteoblastic cell line [14]. Thus, KYD is related closely to the occurrence of osteoporosis.

PMOP is characterised by reduced osteogenesis and enhanced adipogenesis [15]. These changes correlate with reduced trabecular bone volume and increased adipocyte cell size and number [16]. Previous studies have shown that osteoporosis can be induced in mice using a classical method of ovariectomy [17, 18]. These models have been further adapted to establish a KYD model from ovariectomised mice [19, 20]. Jingui Shenqi Pills (JGSQP), a Chinese herbal compound prescription, have been used in TCM for warming and to invigorate the kidney yang [21]. It has been shown to attenuate decreases in the testosterone level and androgen receptor gene expression in mice with KYD [22–24] and to improve the function of damaged ovaries and increase testis telomerase activity [25, 26]. Furthermore, efforts to tonify the kidney yang were shown to more effectively promote osteogenic differentiation and inhibit adipogenic differentiation in bone-marrow-derived mesenchymal stem/stromal cells (BMSCs) than did tonification of the kidney yin [27]. Although these findings suggest interaction between JGSQP and PMOP, little is known regarding the role of JGSQP in pathological bone metabolism. In this study, we sought to better understand the role of JGSQP and its effects on the bone-fat balance in a murine model of ovariectomy-induced osteoporosis with KYD and to evaluate JGSQP as a potential option for the treatment and prevention of PMOP.

## 2. Material and Methods

### 2.1. Experimental Animals and Groups

**Figure 1:**
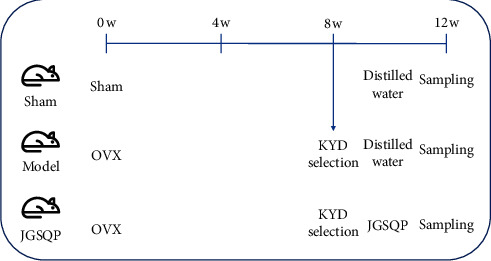
Schematic diagram of the experimental design. w: week; OVX: ovariectomy; and KYD: kidney yang deficiency.

Eight-week-old female C57/BL6 mice (18–22 g) were obtained from the experimental animal center of Guangzhou University of Chinese Medicine (License no. SCXK (Yue) 2018–0034). The mice were raised under conditions of 22–25°C temperature and 25 kPa atmospheric pressure with a 12 h light/dark cycle in the First Affiliated Hospital of Guangzhou University of Chinese Medicine (SYXK (Yue) 2018–0092). Food and water were accessible throughout the experiment. After 1 week of adaptive feeding, the mice were divided randomly into three groups: the Sham group, which received a sham operation in which the fat around the bilateral ovaries was removed; the Model group, which underwent bilateral ovariectomy (OVX) followed by artificial selection of mice with KYD syndrome; and the JGSQP group, which was subjected to OVX and artificial selection of mice with KYD syndrome 8 weeks thereafter, followed by JGSQP treatment for 4 weeks. In the Sham and Model groups, mice were given the same dose of distilled water orally for 4 weeks (Figure 1). All experimental protocols were approved by the ethics committee of the First Affiliated Hospital of Guangzhou University of Chinese Medicine (License no. TCMF1-2019030).

### 2.2. Establishment of the PMOP with KYD Model

The PMOP with KYD model was established as described previously [19, 20]. Briefly, mice were subjected to OVX and allowed to recover for 8 weeks, after which mice exhibiting symptoms of KYD syndrome according to the *Reference Standard for Syndrome Differentiation of TCM Deficiency Syndrome* were selected. Features of KYD syndrome include thin and erect hair, reluctance to move, reduced resistance to scraping, listlessness, unresponsiveness, decreased water consumption, increased sleeping, dark-purple tongue and tail, and dark red eyes. Mice exhibiting three or more of these symptoms were considered to have KYD syndrome.

### 2.3. Preparation of Freeze-Dried JGSQP Powder

Freeze-dried JGSQP powder was prepared as described previously [28]. The single ingredient of JGSQP, conforming to the Drug Standards of National Medical Products Administration of People's Republic of China, was purchased from the First Affiliated Hospital of Guangzhou University of Chinese Medicine (Guangzhou, China). The prescription formula was composed of eight herbs: Processed Radix Aconiti Lateralis (Fuzi, 3.0 g), Cassia Twig (Guizhi, 3.0 g), Dried Rehmannia (Dihuang, 24.0 g), *Dioscorea opposita* (Shanyao, 12.0 g), *Cornus officinalis* (Shanzhuyu, 12.0 g), Alisma Orientalis (Zexie, 9.0 g), *Poria cocos* (Fuling, 9.0 g), and Cortex Moutan (Danpi, 9.0 g). The drugs were soaked in eight volumes of pure water, boiled for 30 min, and filtered. They were then concentrated to 1 g/mL at 80°C and 0.09 MPa. The resulting liquid was then further concentrated by rotary evaporator at 60°C until no droplets remained, frozen at −80°C for 48 h, and lyophilised for 72 h. The resulting powder was stored at −20°C until needed for intragastric administration. The *in vivo* concentration was 0.5 g/mL, representative of the human equivalent dose calculated based on body surface area, consistent with previous studies [28].

### 2.4. Micro-CT

Micro-CT images were analysed as described previously [29, 30]. Briefly, the L4 vertebral bodies were separated, fixed in 4% paraformaldehyde for 24 h, and placed in a rigid plastic tube to ensure that they did not move. Then, the vertebral bodies were analysed using a micro-CT imaging system (SkyScan, Kontich, Belgium) with a 55 kV scanning voltage, 145 mA current, and 4 *µ*m slice thickness. Next, the *μ*CT 80 evaluation programme was used to analyse the volume of interest of the L4 vertebrae. Bone microstructure features were characterised using the following parameters: bone volume/tissue volume (BV/TV), bone surface/tissue volume (BS/TV), trabecular number (Tb.N), trabecular thickness (Tb.Th), trabecular separation (Tb.Sp), and structural model index (SMI).

### 2.5. Bone Histomorphometric Analysis

Histomorphometric analysis of the L4 vertebrae was performed as described previously [30]. Briefly, the L4 vertebrae were fixed in 4% paraformaldehyde for 24–48 h and then placed in ethylenediaminetetraacetic acid (EDTA) decalcification solution for 3–5 weeks. Next, the samples were placed in the distilled water for gradient alcohol dehydration and paraffin-embedded. After trimming of the paraffin blocks, 5-*µ*m-thick slices were cut using a paraffin slicer and visualised by hematoxylin and eosin staining (H&E; Solarbio, Beijing, China). Histomorphometric measurements, including the osteoblast surface ratio (Ob.S/BS, %), number of osteoclasts (Ob.N/BS, 1/mm), adipocyte number/tissue area (mm^2^), and adipocyte area/tissue area (%), were analysed using the Image J software (Wayne Rasband, National Institutes of Health, USA).

### 2.6. RNA Isolation and qRT-PCR

For RNA isolation, 50 mg fresh lumbar vertebrae was snap frozen in liquid nitrogen and ground using a tissue-grinding pestle. Total RNA was then extracted using a MiniBEST Universal extraction Kit (Takara). RNA concentrations and sample purity were assessed using an ultraviolet spectrophotometer (Thermo Fisher). cDNA synthesis was performed using PrimeScript RT Master Mix (Takara). qRT-PCR was performed using SYBR Premix Ex Taq (Takara) in a Bio-Rad CFX96 device for two-step quantitative analysis (40 cycles of 95°C for 30 s, 95°C for 5 s, and 60°C for 1 min). Primer sequences are shown in Table 1. Gene expressions were assessed using the 2^−ΔΔCt^ method.

### 2.7. Western Blot Analysis

Total proteins were extracted from the mice lumbar vertebrae using RIPA lysis buffer (Thermo Fisher) and then quantified using a BCA protein assay kit (Beyotime). Proteins were resolved by electrophoresis on a 10% SDS-PAGE gel then transferred to PVDF membranes (Millipore, Shanghai, China). The membranes were blocked in 5% bovine serum albumin for 2 h at room temperature then incubated in the presence of primary antibodies. Primary antibodies against phosphorylation-AKT (p-AKT; 1:1000; rabbit; ab192623), AKT (1:10000; rabbit; ab179463), BMP2 (1:500; rabbit; ab14933), COL1A1 (1:1000; rabbit; ab34710), FNDC5 (1:1000; rabbit; ab174833), and GAPDH (1:10000; rabbit; ab181602) were incubated for 24 h at 4 °C. The membranes were then washed three times for 5 min each with TBST followed by treatment with a secondary antibody (goat anti-rabbit IgG, 1:3000, ab6939) for another 2 hours at room temperature. Protein levels were evaluated by enhanced chemiluminescence (Bio-Rad, Hercules, CA, USA) following the manufacturer's instructions. The Image J software was used to determine the gray values of the protein electrophoresis bands, which indicates the relative protein expression levels.

### 2.8. Statistical Analysis

SPSS 19.0 (IBM, Chicago, IL, USA) was used for data analysis. All data analysed were quantitative, and comparison among groups was performed by one-way ANOVA followed by Tukey's test for multiple comparisons. *P* values <0.05 were considered to be significant.

## 3. Results

### 3.1. JGSQP Treatment Improved Bone Microarchitecture of Murine OVX-Induced Osteoporosis with KYD

**Figure 2:**
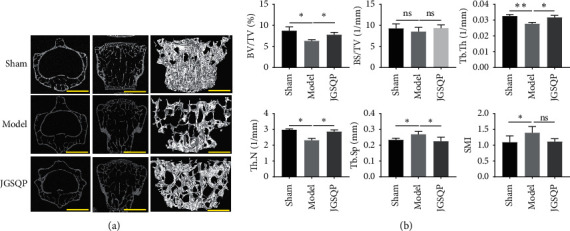
JGSQP improved bone microarchitecture of murine ovariectomy-induced osteoporosis with KYD. (a) Representative 2D and 3D micro-CT images (scale bars = 250 *μ*m); (b) BV/TV, BS/TV, Tb.Th, Tb.N, Tb.Sp, and SMI were calculated based on micro-CT results. Data are expressed as means ± SDs. ^*∗*^*P* < 0.05, ^*∗∗*^*P* < 0.01 (one-way ANOVA with Tukey's multiple comparison test); ns: not significant.

Reconstructed micro-CT images of the L4 vertebrae from the model group revealed reduced, thinning trabeculae and increased Tb.Sp relative to the Sham group. Treatment with JGSQP significantly attenuated damage to the bone microarchitecture. Accordingly, JGSQP treatment created a strong bone-protecting phenotype in mice with OVX-induced osteoporosis and KYD, as evidenced by decreased Tb.Sp and increased Tb.Th, Tb.N, and BV/TV (*P* < 0.05 for all; Figure 2).

### 3.2. JGSQP Attenuated Histomorphological Damage in Murine OVX-Induced Osteoporosis with KYD

**Figure 3:**
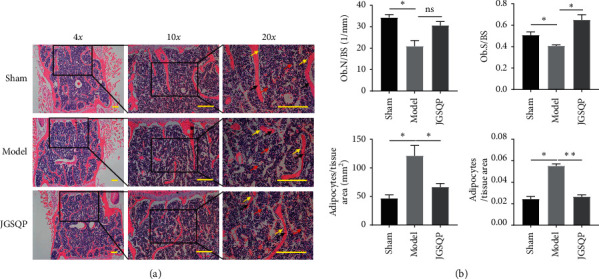
JGSQP attenuated histomorphological damage of murine ovariectomy-induced osteoporosis with KYD. (a) Representative images of H&E staining. (b) The quantifications of Ob.N/BS, Ob.S/BS, adipocyte/tissue area, and adipocyte area/tissue area were calculated based on H&E staining and analysed using the Image J software. Data are expressed as means ± SDs. ^*∗*^*P* < 0.05, ^*∗∗*^*P* < 0.01 (one-way ANOVA with Tukey's multiple comparison test); ns: not significant.

H&E staining of the L4 vertebrae revealed thinner, smaller trabeculae with more lipid droplets and microfractures in the Model group relative to Sham controls. The Ob.N/BS and Ob.S/BS were consistently decreased in the Model group, whereas the adipocyte number and area were increased relative to Sham controls. As before, JGSQ treatment significantly attenuated histomorphological damage in the murine model of OVX-induced osteoporosis with KYD (Figure 3).

### 3.3. JGSQP Reduced Akt and Adipogenesis-Related Gene (*Fndc5*, *PPARγ*, and *Fabp4*) Expression and Promoted the Expression of Osteogenesis-Related Genes (*Wnt10b* and *Osx*)

**Figure 4:**
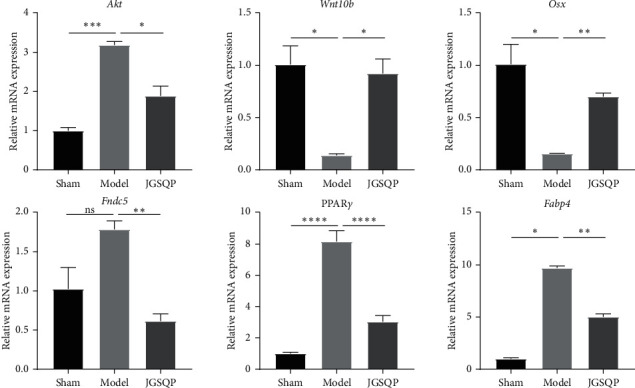
Effect of JGSQP treatment on the mRNA expression of *Akt*, adipogenic (*Fndc5*, *PPARγ*, and *Fabp4*) and osteogenesis-specific genes (*Wnt10b* and *Osx*). Data are expressed as means ± SDs. ^*∗*^*P* < 0.05, ^*∗∗*^*P* < 0.01, ^*∗∗∗*^*P* < 0.001, and ^*∗∗∗∗*^*P* < 0.0001 (one-way ANOVA with Tukey's multiple comparison test); ns: not significant.

Gene expression analyses were conducted using tissues from the L1-L3 vertebrae of all mice (Figure 4). The Model group showed significant downregulation of *Wnt10b* and *Osx* and upregulation of *Akt*, *PPARγ*, and *Fabp4* expressions compared with the Sham group. Although *Fndc5* expression did not differ significantly between groups, a strong tendency toward increased expression was observed in the Model group. After JGSQP treatment, the expression of *Akt* and adipogenesis-related genes (*Fndc5*, *PPARγ*, and *Fabp4*) was downregulated, whereas osteogenesis-related genes (*Wnt10b* and *Osx*) were upregulated.

### 3.4. JGSQP Increased p-AKT, BMP2 Protein Expressions and Reduced FNDC5 Protein Expression

**Figure 5:**
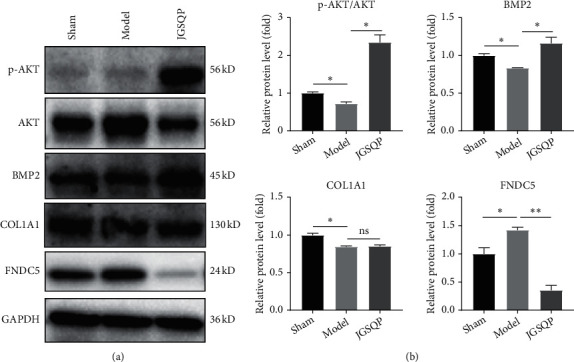
Changes in adipogenesis- and osteogenesis-related protein expressions. (a) Representative images of western blot results. (b) Quantifications of western blot electrophoretograms performed with Image J software. Data are expressed as means ± SDs. ^*∗*^*P* < 0.05, ^*∗∗*^*P* < 0.01 (one-way ANOVA with Tukey's multiple comparison test); ns: not significant.

Protein levels were analysed by western blot. The Model group exhibited significantly reduced p-AKT, BMP2, and COL1A1 expression and significantly increased FNDC5 expression relative to Sham controls. Compared with the Model group, the JGSQP group exhibited significantly greater p-AKT, BMP2 expressions and significantly reduced FNDC5 expression. COL1A1 protein expression did not differ significantly after JGSQP treatment (Figure 5).

## 4. Discussion

An increasing number of studies have focused on the role of bone-fat imbalance in the context of PMOP. Previously, clinical cross-sectional studies showed that bone marrow fat content was positively correlated with the risks of osteoporosis and fracture [31, 32], and other studies have shown that increased bone marrow fat tissue limits the regeneration of damaged bone [16, 33]. Bone and fat interact with each other through endocrine and paracrine forms. Bone marrow fat cells express endocrine factors (ADIPOQ, IGF1, IGFBP2, etc.) and paracrine factors (Wnt10b, BMP4, ANGPT2, etc.) to regulate bone regeneration. Bone secretion factors (OCN, SOST, BMP, PTHrp, etc.) can also regulate fat tissue metabolism [33, 34]. Together, these results show that bone and fat exist in a complex regulatory environment, suggesting that control of the bone-fat balance is important for the prevention and treatment of PMOP.

JGSQP is a TCM prescription used to treat KYD syndrome and many other diseases. It has been shown to play important roles in the regulation of ageing [35], tissue repair [36], and apoptosis [37] and to help prevent acute and critical diseases, including heart failure [38], diabetes [35, 39], asthma [40], neonatal hypoxic-ischemia [41], adrenal insufficiency [42], and hypertension [43–45]. Research conducted using metabolomic and proteomic approaches has demonstrated that JGSQP effectively treats kidney impairment with KYD syndrome involved in Wnt, chemokine, PPAR, and MAPK signaling pathways [36]. Furthermore, tonification of the kidney yang more effectively facilitates osteogenic differentiation and suppresses adipogenic differentiation of BMSCs than does tonification of the kidney yin [27]. However, the effect of JGSQP in terms of bone-fat balance control in an *in vivo* murine model of OVX-induced osteoporosis with KYD has not been reported previously. In the present study, we demonstrated that JGSQP could ameliorate changes to bone microarchitecture in such a model, as confirmed by histomorphological assessment by micro-CT and H&E staining. Furthermore, the quantification of bone histomorphological parameters revealed that JGSQP promoted increases in osteoblast number and surface area while inhibiting such increases in the adipocyte number and area, consistent with the *in vitro* findings of Cheng et al. [27].

To further elucidate the pathogenetic molecular mechanisms possibly underlying these effects, the mRNA and protein expression levels of several genes involved in bone and fat metabolism in the lumbar spine were analysed. *Wnt10b*, *Osx*, and BMP2 are defined broadly as positive regulator of bone formation [46–48], whereas *PPARγ* and *Fabp4* are generally considered to be upregulators of adipogenesis [49, 50]. The role of *Fndc5* in bone formation and adipogenesis remains controversial. *Fndc5* knockout has been shown to block OVX-induced bone loss, suggesting that this gene plays a positive role in adipogenesis [51, 52]. In our study, the bone formation-specific genes *Wnt10b* and *Osx* were upregulated, whereas the adipogenesis-related genes *Fndc5*, *PPARγ*, and *Fabp4* were downregulated in the JGSQP group compared with the Model group, indicating that JGSQP promoted bone formation and inhibited bone marrow lipogenesis, thereby controlling the bone-fat balance in mice with OVX-induced osteoporosis and KYD. In accordance with the results, in protein expressions, the osteogenesis-specific protein BMP2 was increased, whereas the adipogenesis-related protein FNDC5 was decreased in the JGSQP group relative to the Model group.

AKT signaling remains a key pathway that regulates the balance of bone and fat metabolism [53–55]. Increases in p-AKT contributed to the inactivation of *GSK-3β*, which increased downstream *β*-catenin transcription to the nucleus, promoted osteogenic differentiation (e.g., of *Osx*, *Runx2*), and inhibited lipogenic differentiation (e.g., of *Fabp4*, *PPARγ*) [56–58]. In our study, JGSQP activated the expression of p-AKT along with multiple bone formation and adipogenesis-related genes (*Wnt10b*, *Osx*, *BMP2*, *PPARγ*, *Fabp4*, and *Fndc5*), suggesting that it may ameliorate murine OVX-induced osteoporosis with KYD by controlling the bone-fat balance via the AKT pathway. Our investigations demonstrated that JGSQP is an important regulator of the bone-fat balance, and thus that it may be an attractive option for the treatment of PMOP.

## 5. Conclusion

JGSQP treatment reversed murine ovariectomy-induced osteoporosis with KYD through controlling bone-fat balance via the AKT pathway. Thus, this study provides evidence supporting the effectiveness of JGSQP for the treatment of PMOP with KYD.

Although we successfully demonstrated the protective effect of JGSQP treatment on murine ovariectomy-induced osteoporosis with KYD and also gained insight into its underlying mechanism concerning regulating bone-fat balance, this study had several limitations. First, the evaluation of ovariectomy-induced osteoporosis with KYD model lacked precise quantitative index. Second, although some differentially expressed genes were found, advanced techniques such as high-throughput sequencing, gene knockout, and overexpression studies were not employed to explore potentially underlying mechanisms in this study. Third, the findings of this study should be further verified by additional clinical and experimental investigations.

## Figures and Tables

**Table 1:** Quantitative PCR primer sequences.

Gene	Forward (5′-3′)	Reverse (5′-3′)
*Akt*	ATGAACGACGTAGCCATTGTG	TTGTAGCCAATAAAGGTGCCAT
*Wnt10b*	GCGGGTCTCCTGTTCTTGG	CCGGGAAGTTTAAGGCCCAG
*Osx*	AAAGGAGGCACAAAGAAGC	CAGGAAATGAGTGAGGGAAG
*Fndc5*	TTGCCATCTCTCAGCAGAAGA	GGCCTGCACATGGACGATA
*PPARγ*	TCGCTGATGCACTGCCTATG	GAGAGGTCCACAGAGCTGATT
*GAPDH*	ATGTTCCAGTATGACTCCACTCAC	GAAGACACCAGTAGACTCCACGAC

## Data Availability

The data used to support the findings of our study are available from the correspondence author upon request.

## Abbreviations

PMOP: Postmenopausal osteoporosis
OVX: Ovariectomy
JGSQP: Jingui Shenqi Pills
KYD: Kidney yang deficiency
BV: Bone volume
BS: Bone surface
Ob.N/BS: Osteoblast number/bone surface
Ob.S/BS: Osteoblast surface/bone surface
TV: Tissue volume
Tb.Th: Trabecular bone thickness
Tb.N: Trabecular bone number
Tb.Sp: Trabecular bone space
*Osx*: Osterix
Phosphorylation-AKT: p-AKT
GAPDH: Glyceraldehyde 3-phosphate dehydrogenase.
